# A Review of Antimicrobial Resistance in Poultry Farming within Low-Resource Settings

**DOI:** 10.3390/ani10081264

**Published:** 2020-07-24

**Authors:** Hayden D. Hedman, Karla A. Vasco, Lixin Zhang

**Affiliations:** 1 Illinois Natural History Survey, Prairie Research Institute, University of Illinois Urbana-Champaign, Champaign, IL 61820, USA; 2 Department of Microbiology and Molecular Genetics, Michigan State University, East Lansing, MI 48824, USA; vascokar@msu.edu (K.A.V.); lxzhang@epi.msu.edu (L.Z.); 3 Department of Epidemiology and Biostatistics, Michigan State University, East Lansing, MI 48824, USA

**Keywords:** antimicrobial resistance, intensive poultry production, economic development, food security

## Abstract

**Simple Summary:**

Poultry production can function as an instrument for poverty alleviation and economic development. As low-income countries transition into higher incomes alongside growing urban populations, there will be an increasing demand for quality sources of animal products. Consequently, poultry production systems will continue to shift from subsidence agricultural practices to intensive food production that implies routine antimicrobial usage. Promotion of intensive poultry production could increase antimicrobial resistance (AMR) within resource-limited settings lacking in effective biosafety and biosecurity measures. Bacterial resistance lessens the portfolio of antimicrobials available in poultry husbandry and potentially human medicine. This issue requires a systems framework in order to evaluate the various social and biological factors driving the emergence of resistance within the context of intensive poultry production.

**Abstract:**

The emergence, spread, and persistence of antimicrobial resistance (AMR) remain a pressing global health issue. Animal husbandry, in particular poultry, makes up a substantial portion of the global antimicrobial use. Despite the growing body of research evaluating the AMR within industrial farming systems, there is a gap in understanding the emergence of bacterial resistance originating from poultry within resource-limited environments. As countries continue to transition from low- to middle income countries (LMICs), there will be an increased demand for quality sources of animal protein. Further promotion of intensive poultry farming could address issues of food security, but it may also increase risks of AMR exposure to poultry, other domestic animals, wildlife, and human populations. Given that intensively raised poultry can function as animal reservoirs for AMR, surveillance is needed to evaluate the impacts on humans, other animals, and the environment. Here, we provide a comprehensive review of poultry production within low-resource settings in order to inform future small-scale poultry farming development. Future research is needed in order to understand the full extent of the epidemiology and ecology of AMR in poultry within low-resource settings.

## 1. Introduction

Antimicrobial resistance (AMR) remains a growing threat for human and animal health, lessening the ability to treat bacterial infections and furthering the risk associated with morbidity and mortality caused by resistant bacteria. Ensuring the effectiveness of antimicrobials to treat bacterial infections remains a pressing issue for both veterinary and human medicine [1,2,3,4]. The connection between antimicrobial use (AMU) and selection for resistance has been extensively studied [4,5,6]. Studies have widely documented agricultural AMR emergence leading to resistance in clinical settings [7,8,9,10,11,12,13]. Within the United States, 80% of antimicrobial agents produced are applied to animal production; [14] and globally over 70% of global antimicrobials produced on Earth are used in food-animal production [7,15,16]. Although the European Union has banned the use of antibiotics for growth promotion, regulation of growth promotion antibiotics is sparse throughout the world [8]. Therefore, intensive animal food production can lead to the selection for the emergence of resistance due to the extended use of antibiotics for growth promotion, disease prevention, and infection treatment [17,18,19].

**Figure 1:**
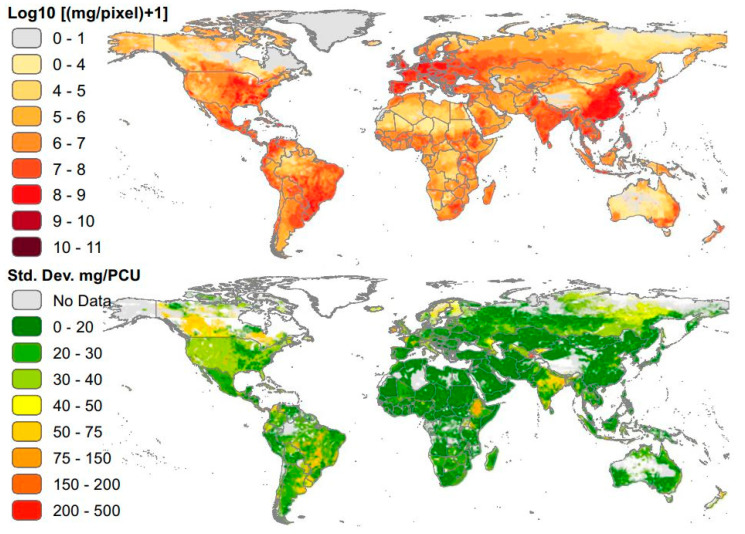
Global antimicrobial consumption in livestock in milligrams per 10 km^2^ pixels (Top) and average SD of estimates of milligrams per population correlation unit (PCU) (Bottom) [26].

**Figure 2:**
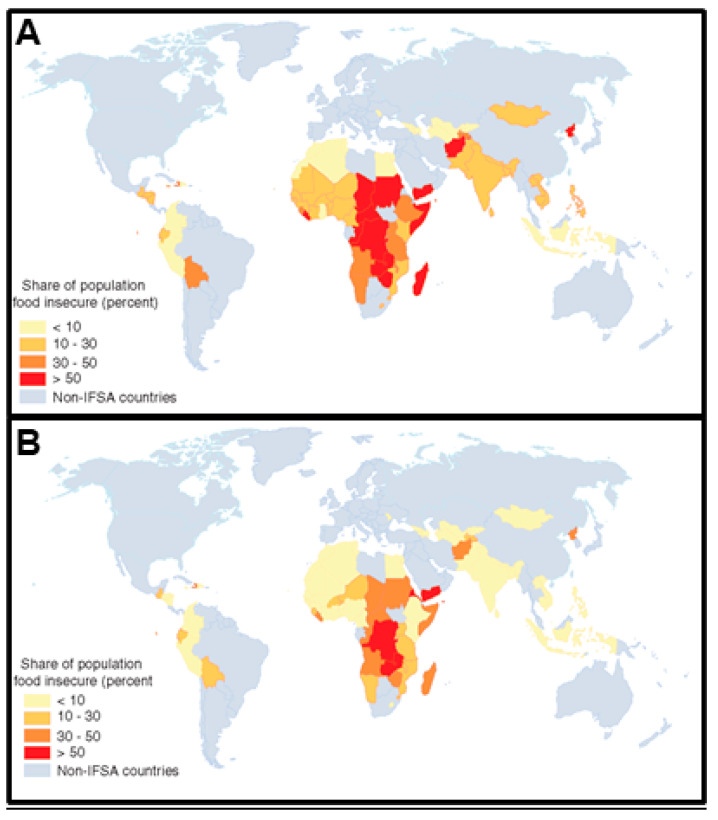
(**A**) Share of population that was food insecure in 2019 and (**B**) projected to be insecure in 2029. Grey corresponds to regions where data is unavailable [31].

**Figure 3:**
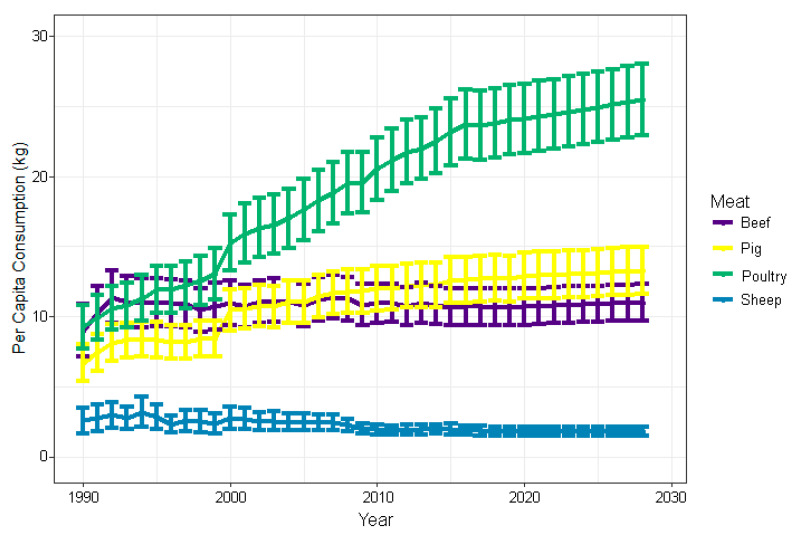
Predicted trends 1990–2028 in global livestock meat per capita (kg) consumption. Data summarized from OECD meat consumption [32].

Animal agriculture within low-resource settings is of high importance as many countries transition to more intensive animal farming practices, leading to greater AMU and thus an intensified risk of AMR exposure to animals and humans worldwide (Figure 1). As low- to middle income countries (LMICs) continue to transition to high incomes, there will be a continuously increasing demand for quality sources of animal protein [20,21,22,23,24] (Figure 2). Food and Agriculture Organization (FAO) reported a global increase egg production and of poultry meat and worldwide, with a total of 87 million tons of eggs and 123 million tons of poultry meat (37% of meat production) in 2017. As food animal production rapidly expands, as well as the antimicrobial use, it is important to evaluate global trends of AMR emergence associated with poultry production [7,15,25] (Figure 3). It is estimated that agricultural intensification will lead to an increase of 67% in antimicrobial usage by 2030, predominantly led by LMICs [26]. For instance, China, where 50% of global pork production originates from, is expected to consume 30% of veterinary antimicrobials sold in 2030 [25]. From 2000 to 2010, antibiotic use in 71 countries increased by over 36% with Brazil, Russia, India, China, and South Africa (BRICS) attributing to over 75% of the increase [27,28] As countries continue to develop, antibiotic use in many LMICs has already converged (and exceeded) that of levels observed in high-income countries (HICs) [29]. Rising income is a major driver of increased AMU in LMICs [30]. Currently, selected LMICs exhibit AMU rates that surpassed those of HICs. Moreover, it is predicted that soon the AMU rates of majority of LMICs will surpass those of HICs [29]. 

In LMICs, AMU increased by 65% from 2000 to 2015 [29]. The most common antimicrobials applied to food animal production include tetracyclines, sulfonamides, and penicillins [33]. A systematic review evaluating AMU in food production reported 51 of the most commonly administered antimicrobial agents in aquaculture and animal agriculture, 39 (or 76%) are frequently used in human medicine, and 6 antimicrobial classes encompass the WHO CIA list [34]. Given that poultry production comprises a substantial portion of global food production and AMU, it is important to address evident rising use of antibiotics administered in poultry farming in order to improve antimicrobial stewardship.

Poultry, one of the fastest per capita produced livestock [35,36] (Figure 3), will continue to expand as countries shift from subsistence to intensive farming that also requires routine AMU [37,38]. In comparison to other terrestrial livestock, the ubiquity of poultry is attributable to several key characteristics: small body size, relatively short life cycle, high energy uptake efficiency, and robust adaptability to environmental conditions [39,40,41]. Poultry is defined as a group of domesticated birds raised for animal products (e.g., meat, eggs, manure), fiber (e.g., feathers), entertainment (e.g., racing, exhibition, hunting, etc.), or work (e.g., messenger pigeons). Most poultry species encompass a few avian orders that include Galliformes (chickens, turkeys, quail, pheasants, grouse, guinea fowl), Anseriformes (ducks, geese, swans), and Columbiformes (pigeons and doves), and Ratites (ostriches, emus) [41,42,43]. Poultry is one of the fastest growing per capita meats produced in the world [36,44,45] (Figure 3). In the last half century, the global poultry annual growth rate was 5%. Contrastingly, it was only 1.5% for beef, 3.1% for pork, and 1.7% for small ruminants [43]. Chickens (*Gallus gallus domesticus*) comprise of 90% of global poultry production, amounting for approximately 23 billion chickens [46]. 

Foundational reviews have suggested that there is not a global standard in biosecurity practices for small-scale poultry farming [15,47]. More importantly, AMR remains critically understudied within the context of LMICs [48,49]. In resource-limited settings, which comprise the majority of LMIC poultry farming systems, poultry production commonly occurs among small-scale, family operations with limited biosecurity due to constraints in hygiene and sanitation [50,51,52,53]. Additionally, to facilitate economic growth, developmental organizations often promote the intensive small-scale poultry farming [39,54,55]. These interventions can lead to the potential risk of promoting AMR transmission to other domestic animals, wildlife, and surrounding human populations. 

This review aims to provide a systems lens of the major drivers of AMR in poultry farming within the context of low-resource settings in order to inform future veterinarian and public health policy and implementation. Therefore, this paper will first provide introduction of AMR evolution and spread, followed by description of the origins of AMR in poultry, and then an overview of poultry production systems, thereafter, an evaluation of small-scale poultry development, and concluding with barriers to improved antimicrobial stewardship programs in low-resource settings. Our review provides a novel eco-epidemiological framework for assessing the impacts of intensive poultry farming within low-resource setting.

## 2. Mechanisms for Antimicrobial Resistance Spread and Evolution

AMR bacteria are naturally found in the environment because many antibiotics are produced by other organisms such as fungus (e.g., penicillin) and soil bacteria (e.g., streptomycin, chloramphenicol, and tetracycline) [10]. In many cases, bacteria exhibit intrinsic resistance across an entire species like in the case of macrolide resistance in *Escherichia coli* [56]. Since introduction of almost every novel antimicrobial, evolved bacterial resistance has shortly followed [57]. Typically, it takes antibiotic development at least 10 years before certification for general public us [58,59]. In contrast, bacteria can evolve resistance within a few hours [60], making the evolutionary arms race a one-sided competition. As AMR continues to pose threat to public health and animal health, a clear understanding of mechanisms leading to the development of AMR remains essential for monitoring AMR emergence and dynamics among varying host population species [4,61,62,63]. 

Acquired bacterial resistance is caused by four general mechanisms including inactivation, target alteration, decreased permeability, and increased efflux [64]. First, target site changes typically occur from spontaneous mutation of a bacterial gene with selection pressure of antibiotics [36]. Two examples consist of mutations in RNA polymerase and DNA gyrase which facilitate resistance in rifamycins and quinolones, respectively [65]. Second, target alteration uses a strategy to make the antibiotic ineffective through enzymatic degradation, commonly occurring among aminoglycosides, chloramphenicol, and beta-lactams [66]. Third, Gram-negative bacteria can decrease permeability to selectively filter antibiotics from entering the cell membrane [67]. Fourth, efflux pumps function mainly to release toxic substances from the bacterium and many of these pumps can transport an extensive variety of compounds [68,69].

**Figure 4:**
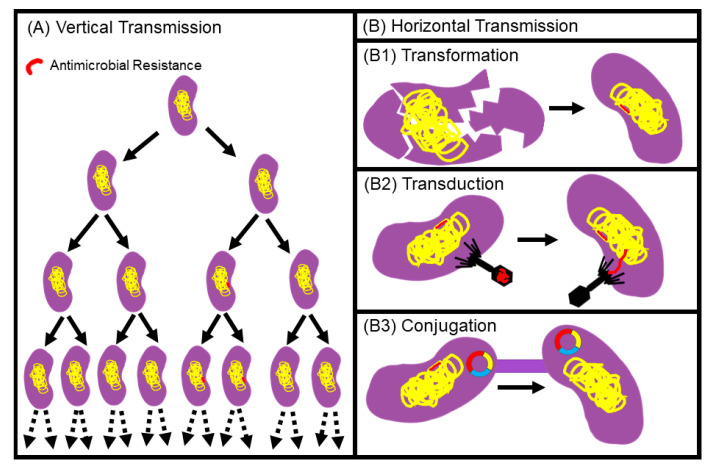
Primary pathways involved in the exchange of genetic information conferring antibiotic resistance consisting of (**A**) vertical transmission and horizontal transmission (**B**).

Two fundamental biological pathways that facilitate the evolution and dissemination of resistance include vertical gene transfer (VGT) and horizontal gene transfer (HGT) (Figure 4). First, resistance can occur among a pre-existing phenotypic-resistant bacteria population. Genetic mutations within bacterial genome that promotes AMR can be transferred from parent to daughter cells, via VGT, such as the resistance to fluoroquinolones and oxazolidinones [70,71,72] (Figure 4A). In the second pathway, genetic mechanisms facilitating resistance can be exchanged between bacterial species, which is also often described as horizontal gene transfer (HGT) [73] (Figure 4B). HGT usually manifests through the following three mechanisms: (1) transformation, defined as the exogenous DNA from environment through cell membrane, (2) transduction, defined as gene transfer from one bacterium to another through a viral medium, and (3) conjugation, defined as gene transfer from a donor to a recipient cell through direct cell-to-cell contact mediated by plasmids [73,74]. Transformation and transduction usually occur between microorganisms that are closely phylogenetically related. Whereas, conjugation can occur between different Phyla allowing a promiscuous bacterial transfer of AMR. Plasmids are the most important medium of antibiotic-resistant gene (ARG) dispersion. These circular DNA structures (plasmids) are often scaffolds of ARGs and mobile genetic elements (MGEs) (e.g., transposons, integrons, and insertion sequences), facilitating the emergence of multidrug-resistant (MDR) bacteria [63,75,76,77]. 

## 3. Context for Antimicrobial Use in Poultry Production

The discovery that antimicrobials fed in subtherapeutic concentrations to poultry expedited their growth was accidental [78]. In 1946, the first recorded use of antimicrobial growth promoters (AGPs) was documented in chickens [78]. Soon after, farmers in post-war United States and Europe were struggling to supply for an increasing demand for poultry food products [25]. Meanwhile antimicrobials administered for growth promotion and disease prevention became a vital component for intensive poultry production [79,80,81], leading to a novel model for industrial poultry systems that would be later replicated among LMICs [82]. In 1951, the United States Food and Drug Administration (FDA) approved delivery of antimicrobial agents in feed without veterinary prescription [83]. Meanwhile, approval for antimicrobial use in animal feed varied among European nations [25]. In 1970, the Council directive 70/534 standardized European policy related to feed additives in food production [1]. In 2006, European Union regulation No. 1831/2003 limited use of antimicrobials for animal nutrition beyond treatment of coccidiostats and histomonostats [25]. In 2013, Under Guidance for Industry (GFI) #213, the FDA restricted use of AGPs in animal production that are important for human medicine [83]. Subsequently, in 2014, the Canadian government modeled its ban on select AGPs based off of the FDA policy [84]. Various Organization for Economic Co-operation and Development (OECD) countries have instituted bans on APGs (e.g., Mexico, South Korea, New Zealand), while APGs remain authorized in other countries (e.g., Japan) [25]. AGPs are not ban in most non-OECD countries, which comprise of some of the leading poultry producers including China, Brazil, Russia, Argentina, India, Indonesia, Philippines, and South Africa [28]. 

Determining productivity gains of AGPs at a global scale remains extremely difficult due to the lack of availability of quality data outside of a few HICs [25]. Bans on AGPs exhibit minimal economic impacts among optimized production systems within HICs but potentially greater impacts among lower income countries where there is less developed biosecurity and sanitation practices [25]. Restrictive policies on the use of antimicrobials in LMICs could potentially increase animal disease burden where antimicrobials also serve as a substitute for quality hygiene and sanitation [17]. Furthermore, select antibiotics used in poultry farms are important for animal health including tetracyclines, aminoglycosides, lincosamides, amphenicols, fluoroquinolones, sulfas, and beta-lactams [31]. Judicious review of the usage of AGPs is necessary for maintaining effective antimicrobial stewardship worldwide. 

## 4. Introduction to Poultry Production Systems

### 4.1. Large-Scale Intensive Poultry Production

**Figure 5:**
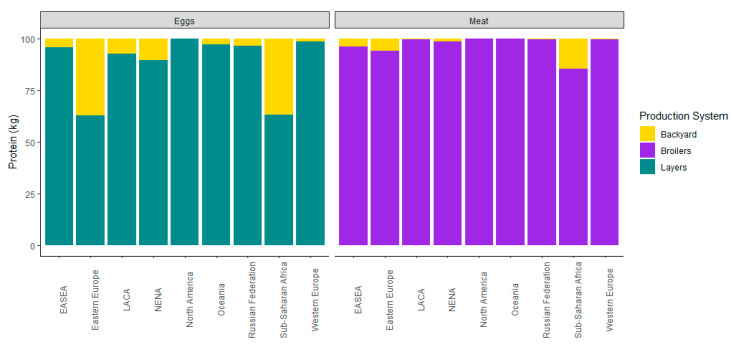
Poultry egg and meat production by husbandry type and geographic region (EASA: East Asia and Southeast Asia; LACA: Latin America and Caribbean; NENA: Near East and North Africa). Data summarized from FAOSTAT Statistical Database [46].

The use of antimicrobials in intensive poultry production is becoming increasingly common at smaller scales within low-resource settings because of its high throughput of meat and egg products [43,85,86,87]. As urban populations continue to rise among LMICs, the demand for animal-source products will increase [29,88,89]. Defining characteristics of intensive large-scale farming include confined hatchery environments that house chickens at high densities (>1000), routine AMU [86], and breed selection of predominantly broiler chicken for meat production and layer chicken for egg production [90] (Figure 5; Table 1). Because of AGPs the broiler chicken is considered the most resource efficient livestock [90,91], leading to over 50% increase in body mass from 1955 to 1995 while substantially lowering the feed and time required [92]. 

It is important to highlight the varying risk factors associated with large-scale farms in relationship to smaller, family operated operations. Within the context of LMICs, AMR exposure from commercial farms is generally localized to occupational exposure or to animal fecal environmental contamination of AMR pollutants to surrounding soil or water runoff [10,94,95]. Consequently, these intensive systems should be isolated from dense human concentrations and ecological sensitive landscapes. Several countries established minimum distances between farms, water courses and human populations [96,97]. In LMICs, large-scale farms present within densely populated peri-urban and urban settings can function as potential hotspots for zoonoses and MDR bacteria [98,99,100,101].

### 4.2. Family Poultry Husbandry

Family managed farming operations are found throughout the world and make up the majority of global poultry production [39] (Figure 5). “Family poultry” is used to broadly define household, small-scale poultry production systems present in rural, peri-urban, and urban environment that provide subsidence or income [39,43,102]. The Food and Agriculture Organization (FAO) of the United Nations (UN) has categorized family poultry operations into four major subgroups: small extensive, extensive, semi-intensive, and intensive [15]. These four types of operations vary by inputs, outputs, gender dimensions, chicken breeds, biosecurity and biosafety, and environmental impacts [15,103] (Table 2). This series of family operated poultry systems can appear on a continuum, yet it is critical that families have access to husbandry practices that reflect their own farming capacity and objectives [15,103]. This framework provides a context for discussing variable biosecurity risks related to drug resistance associated with poultry husbandry in LMICs. 

Small-extensive and extensive scavenging poultry farming typically involve local breeds of poultry that can be characterized with a variety of terms ranging from “village”, “indigenous”, “backyard”, or “household” [104,105]. For the purpose of this review discussion, we will use “backyard” to be consistent with terminology used by FAO [15]. Backyard chickens, generally raised without routine antimicrobial therapy, can function as both a regular source of marginal income or, like a liquid asset, sold during times of need to purchase food or medical supplies [55]. Small-extensive and extensive farming systems are generally managed by female heads of households and children, supporting agency to women to make important decisions regarding household economics [106,107,108]. Despite the small flock sizes, scavenging system accounts for approximately 75% of poultry operations among LMICs across Asia and Africa [40,109,110]. Studies have found that occurrence of extensive poultry production system is strongly associated with urbanicity and resourcefulness of regions [111,112]. Although backyard breeds vary phenotypically and genotypically by regional geography [39], their primary function as open foragers within scavenging farming systems is universal [39,43,105]. In contrast to industrial broiler and layer chickens, backyard chickens lack artificially selected genes for high resource efficiency [92] [Table 1]. However, in comparison to commercial chicken breeds, backyard chickens that are adapted to local environments generally yield higher survival to environmental pressures such as predators, infectious diseases, or natural disasters [110,113,114,115].

In contrast to small-extensive and extensive scavenging poultry farming, semi-intensive and intensive family operations apply similar practices as intensive animal production systems at the household level [15]. Small-scale intensive operations typically raise broiler or layer chickens with antimicrobials administered in commercial feed and water [15,43]. Various studies have reported that inappropriate use of antimicrobial agents remains common among family operated systems due to a lack of AMR awareness and access to quality veterinary services [116,117,118]. Intensive family operations typically lack the financial resources to support minimal biosafety standards that are present in commercial operations. Some of these shortcomings in biosafety can include lack of personal protective equipment, inadequate sanitation, and unsafe manure disposal [47,119]. Moreover, poultry chain (production to consumption) usually occurs within household enclosure [120,121]. These settings can function as high risk environments for occupational exposure resistant bacteria and ARGs because of the frequent and intimate contact between animals (both poultry and other non-poultry domestic) and members of the household [54,103,122]. Currently, there lacks an international standard that incorporates biosecurity measures of backyard chicken farming [47]. Consequently, many studies have pointed to intensive small-scale animal husbandry as a high-risk practice that can potentially lead to regional outbreaks and global disease pandemics [54,123,124]. 

## 5. Small-Scale Poultry: An Instrument to Sustainable Development

UN launched the 2030 Agenda for Sustainable Development to promote peace and prosperity for all people and the planet both present and in the future. This agenda established 17 Sustainable Development Goals (SDGs) for mitigating impacts of human pressure on the planet that has led to planetary crises such as biodiversity loss, climate change, environmental degradation, and negative impacts on health and nutrition. Studies have acknowledged the interrelatedness nature of the SDGs [125,126,127]. Furthermore, integrative solutions are essential to effectively manage agricultural intensification, land-use change, and gender equity [103,107,128]. Unfortunately, public health implementation programs that target specific SDGs might have unforeseen negative impacts on other goals [129,130]. It is critical that policy is rooted in common denominators of SDGs in effort to prevent emergence of unwanted negative consequences. 

Although poultry development has existed for decades, the recent UN SDGs have stimulated increased development projects centered around poultry husbandry; many of the SDGs overlap within the context of small-scale poultry husbandry [43,55] (Table 3). These programs are typically led by developmental agencies, international agencies, and non-governmental organizations that collectively invested in supporting small-scale poultry at the family or community level. Within the last few decades, small-scale poultry development programs have been widely implemented as a means to promote economic stability, especially among resource-limited settings [43,103]. Low input and output costs have facilitated the promotion of poultry production in LMICs [39,55]. 

There have been numerous studies highlighting how small-scale poultry facilitates improved economic stability, increased food security, and gender equity [39,43,55]. For example, researchers in Mozambique reported that village poultry provided an essential role through poverty alleviation and economic stability among rural populations burdened with the impacts of HIV/AIDS [131]. In Bangladesh, the Department of Livestock Services (DLS) and the Bangladesh Rural Advancement, Committee (BRAC) scaled down large poultry operation models and appointed women groups as managers [107]. This successful intervention was later adapted and applied to other countries including Malawi [106] and South Africa [132]. In another example, Network for Smallholder Poultry Development Program (NSDP) has initiated projects throughout West Africa and Asia. NSDP provides a cross-sector approach by developing capacity through training various sectors with women groups, local poultry vendors, private veterinarians, community educators, and trained village vaccinators [42].

## 6. Potential Risk of Antimicrobial Resistance Human Exposure Associated with Small-Scale Poultry Development

Despite the positive outcomes of poultry development, there are many constraints associated with local stakeholder poultry production that need to be acknowledged. These constraints include various predators, nutrition quality, genetic breeding, training and management, infrastructure and capital, farmer organization, governmental policies, and most relevant to public health are the associated biosafety and biosecurity risks [55]. Moreover, the shortcomings in biosafety management is commonly addressed with increased use of antimicrobial agents, facilitating higher burdens of MDR bacteria in small stakeholder livestock production [54,122,133]. 

Unfortunately, applying intensive poultry farming to low-resource settings can potentially exacerbate pre-existing health burdens [54]. Small-scale poultry development can function as a double-edged sword without proper oversight, inadvertently exacerbating poverty and food insecurity. Commonly, development programs source poultry from commercial confinement operations [7,86,134]. This intervention approach could risk exposing family members and surrounding community members to potential AMR bacteria and zoonoses [122]. Distinct from large commercial-scale production, currently, no international biosecurity standards exist for family managed poultry operations [135,136]. In resource-limited environments, these husbandry operations occur near (or within) households; these environments can potentially lead to increased AMR spread due to poor water sanitation, inadequate hygiene conditions, and intimate human–animal interactions [24,30,122]. Furthermore, these environments might increase the probabilities of anthropo-zoonotic transmission of AMR, meaning the transmission of AMR from humans to animals, which could be a route for the spread of resistance to antibiotics non-commonly used in poultry farming in LMICs such as fluoroquinolones and colistin [47,137].

The spreading of AMR and infectious diseases in small-scale productions is also related to live-animal markets where different animal species are sold to local farmers. The convergence of humans and animal species coming from several locations provides a unique opportunity for infectious agents to jump species and propagate. Poultry sell in wet markets usually come from intensive operations with a wide AMU. As a result, cases of 1-day old chickens harboring multidrug-resistant bacteria have been reported in LMICs [76,138,139,140,141].

Intensive small-scale poultry systems raise larger flock volumes, which can lead to potentially higher economic yields [43]. Men of household that were previously disengaged from poultry husbandry are more likely to take control of husbandry management once the practice becomes lucrative [142]. Interventions that foster intensive production could indirectly facilitate gender inequity as increased incomes incentivize men to take over flock management [103]. Larger operations have shown shifts in gender distribution as they are usually managed by men [143]. These cascading effects highlight the necessity to integrate gender dimensions within decision making of poultry development. 

## 7. Eco-Epidemiology of Poultry Production: A Framework for Evaluating Antimicrobial Resistance of Poultry Origin in Low-Resource Settings

**Figure 6:**
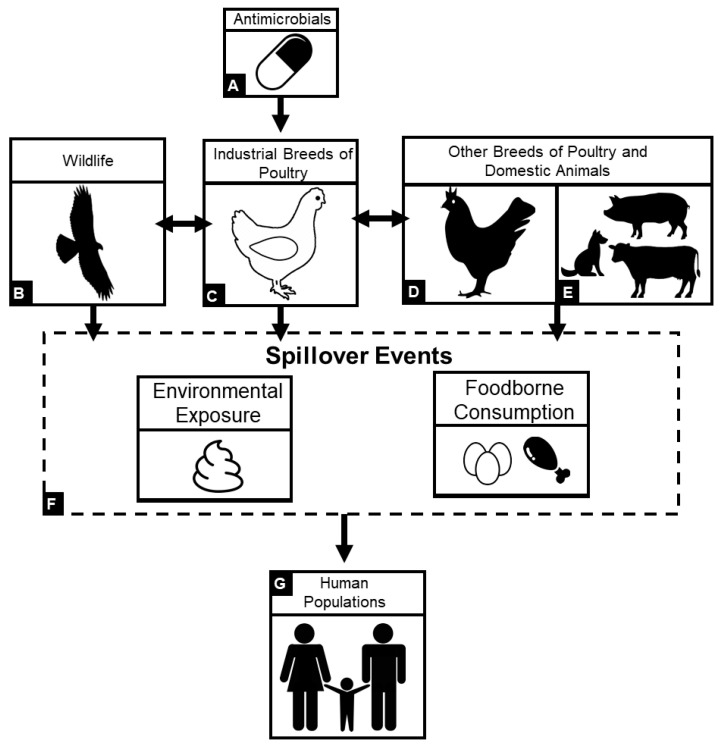
Conceptual graphic illustrating antimicrobial resistance associated with intensive poultry production.

The increasing global burden of resistance challenges experts to design innovative interventions to disentangle the complex social and ecological dimensions that facilitate the evolution, spread, and persistence of AMR. This effort stems from Boulding’s Skeleton of Science [144], demonstrating multiple levels of disciplines are essential for holistic research, and this framework can be applied to the context of AMR as it has been applied to other emerging infectious diseases (EIDs) [145,146,147]. Here, we apply a systems framework for evaluating the eco-epidemiology of AMR associated with poultry production (Figure 6; Table 4).

We recognize that the presence of AMR determinants is an important driver in the dynamics of bacterial resistance in poultry production. It is important to acknowledge that selection for resistance is heavily influenced by variability in antimicrobial administration, which can include antimicrobial classification, duration of therapy, and Defined Daily Dose [159,160,161,162]. Studying ecological indicator species that are present within the avian microbiota can serve to inform the status of bacterial populations sensitive to dynamical changes of AMU in poultry husbandry [163,164,165]. In particular, multiple other literature reviews evaluating AMR bacteria from poultry have given priority to foodborne, specifically, *Escherichia coli*, non-typhoid *Salmonella* spp., and *Campylobacter* spp. because of their importance to veterinarian medicine and public health [7,166,167,168]. Commensal bacteria can potentially be a public health threat because it can transfer ARGs to human microbiota and eventually to human pathogens [73,169]. There are many other pathogenic and opportunistic pathogens associated with poultry, surveillance of poultry microbiome and resistome can provide a better insight in the ecology and abundance of AMR microbial hosts. 

Furthermore, mechanisms facilitating the emergence of resistance within the avian microbiota vary by poultry farming practices [170,171,172,173]. It is extensively documented that poultry production operations applying routine use of antimicrobials have a higher potential risk for the selection of AMR bacteria compared to antimicrobial-free operations [119,139,141,174,175,176,177,178,179,180]. This polarity in antibiotic therapy is increasingly common in LMICs where there is a demand to support growing urban populations alongside preserving traditional poultry husbandry practices [39,43]. International development programs have promoted small-scale intensive poultry farming as an effort to alleviate poverty [55,107]. Intensive agricultural interventions lacking in biosecurity measures and financial support could further burden families with increased risk of zoonotic infections and AMR [122,133,181,182]. Additionally, poultry intensification can have cascading socioeconomic impacts on farming communities such as facilitating shifts in gender demographics of poultry management or local market fluctuations depending on cultural appropriateness of poultry products [39,103]. It is imperative to analyze the emergence of AMR within low-resource settings using a systems framework to effectively address the many interactive layers. 

### 7.1. Antimicrobial Resistance Transmission to Other Domestic Animals and Wildlife

There is a gap in the literature evaluating the epizoology of resistant bacterial populations spread from poultry to other animals. In particular, intensively raised poultry can serve as reservoir hosts for AMR bacteria [95,160] (Figure 6; Table 4). Local breeds of free-ranging backyard chickens are likely sentinel hosts for AMR carriage from intensively farmed poultry because they are typically housed in the same settings [152,176,183,184,185]. Shared husbandry environments, combined with limited sanitation and the implementation of intensively farmed poultry, impact the microbiota of backyard chickens. In Ecuador, village-scale introduction of intensively raised broiler chickens facilitated a high increase in beta-lactamase CTX-M-producing *E. coli* in backyard chickens [141]. Within the same study region, Hedman and colleagues observed that over time phenotypic *E. coli* resistance profiles of backyard chickens and children mirror changes of AMR profiles of intensively raised broiler chickens [139].

In addition to AMR exposure between breeds of chickens, intensive poultry farming has been linked to AMR transmission between various domestic and wild animal species [186]. Bacterial strains are more likely to transcend host species barriers and colonize novel animal hosts that share an overlap in ecological niches or close evolutionary relatedness [187]. Smith (1970) documented one of the earliest reports of AMR Enterobacteria species spreading between chickens, cattle, and swine [188]. Among larger food production facilities, there is an increased risk of exchange of AMR bacteria and genetic elements between livestock and poultry [189,190,191,192,193]. Phylogroups of resistant *E. coli* reveals that chickens, pigs, and cattle demonstrate are very similar with respect to their AMR phenotype [194]. Moreover, there appears to be an increased risk for AMR spread among animal production centers that house multiple species, especially with poor biosecurity measures. In Nigeria, poultry and cattle operations exhibited shared sources of manure contamination with abundant MDR and virulent *Enterococcus* spp. [150]. In Costa Rica, AMR transmission from intensive small-scale poultry to neotropical avifauna were reported in *E. coli* isolates resistant to tilmicosin, tetracycline, ampicillin, amoxicillin with clavulanic acid, ticarcillin, cephalothin, and ARGs corresponding to tetracycline resistance [154]. Similarly, unhygienic disposal of poultry carcasses was documented in quinolone resistance in avian scavengers [195]. Furthermore, cross-species transmission of AMR can be facilitated by rodent and insect vectors that frequently occupy intensive poultry husbandry settings [48,55,196,197,198,199,200,201,202]. Evaluation of AMR bacteria from poultry, in addition co-occurring animals, can comprehensively strengthen surveillance efforts.

### 7.2. Zoonotic Antimicrobial Resistance Transmission 

AMU in poultry production contributes to the dissemination, selection, and persistence of AMR in human populations. Understanding the primary transmission routes of zoonotic bacterial resistance is critical for veterinary medicine and public health. Resistant bacteria of avian origin that have the potential to colonize human microbiota is not a novel concept [203]. Studies have widely demonstrated that there is a strong occupational exposure risk of zoonoses for commercial poultry workers [94,156,204,205,206,207,208,209] (Figure 6; Table 4). In addition, there is a growing body of research suggesting that families engaged in poultry husbandry exhibit an increased risk for carriage of AMR bacteria and diarrheal pathogens [210,211,212,213,214,215,216,217,218]. Also, AMR bacterial carriage in humans via foodborne transmission remains a risk in resource limited settings that lack biosafety regulations in abattoir or retail markets [50,219,220,221]. Clinical reports of AMR carriage in humans have also been linked to poultry products [222,223,224,225]. For instance, in China, a longitudinal study evaluating poultry practices using whole-genome sequencing has detected the co-existence of *bla*_CTX-M_ and *mcr-1* in ESBL-producing *E. coli* of avian and human origin; further phylogenetic analysis revealed close relatedness, which suggests an active transmission of ESBL-producing *E. coli* and *mcr-1* in both clinical medicine and veterinary medicine [226]. Furthermore, a study of MDR *E. coli* from a rural community in Ecuador, found that livestock (including poultry), companion animals and humans shared similar AMR profile; however, genetic analysis revealed that ARGs were located on different plasmid structures and bacterial strains, revealing that HGT plays a significant challenge for understanding the movement of AMR in a community [227].

In many regions of the world, small-scale poultry operations are connected to vast market networks centralized in urban live markets. These markets can pose an enormous threat for AMR and zoonoses emergence since poultry are housed and later slaughtered within the same setting of other domestic animals and wildlife [228,229,230].

### 7.3. Poultry Waste Management and the Environmental Resistome

Poultry production generates large volumes of excretion that comprise of solid waste and wastewater. Primarily, solid waste consists of litter (a mixture of bedding substrate, excreta, feed, feathers, shells), abattoir waste, and carcasses whereas wastewater usually comes from disinfecting and washing hatchery and abattoir environment [231,232,233,234,235]. Manure makes up the most abundant waste product. In many cases, manure administration can provide a nutrient-rich source of fertilizer or livestock feed supplement due to nitrogen (3.3% NO_3_), phosphorus (3.4% P_2_0_5_), and potassium (1.7% K_2_O) for crop fertilizer and recognized as the best organic fertilizer collected from terrestrial food animals [231]. Throughout the world, poultry litter is also recycled as animal feed [236]. The aqueous leachate of poultry litter is toxic to many organisms, leaching of nutrient inputs into aquatic systems can facilitate eutrophication and algal blooms [237]. Despite the applications of poultry byproducts, it is widely noted that poultry fed antibiotics can then shed AMR bacteria and ARGs into the soil environment [10,94,238]. The majority of antimicrobials used in animal husbandry retain activity after renal or biliary excretion [88]. Furthermore, it is critical to mechanisms that determine the fate of environmental resistance. 

Within the context of LMICs, fecal contamination has largely been attributable to the emergence of pathogenic and AMR bacteria across all scales of poultry production [239,240,241]. Meanwhile, other indirect pathways may include vectors such as aerosols, dusts, insects, rodents, humans, and other domestic animals that come into contact with fecal particulates [10,242,243] (Figure 6; Table 4). Regional climate plays a crucial role in furthering the spread of AMR pollutants, especially among tropical landscapes that are subject to extreme flooding events [244,245]. Poultry production operations that utilize untreated water could subsequently expose poultry to resistant bacteria [246]. These various pathways can also contribute to spread of resistant determinants into soil, surface water, ground water, and agricultural crops [247]. 

In LMICs, poultry litter is typically locally disposed within the community landscape as either livestock feed or crop fertilizer [248,249]. Even in the absence of ongoing poultry production, localized manure disposal can present public health challenges; in addition to AMR determinants [196,247], poultry litter may elevate the concentration of metals such as zinc and copper that are commonly associated with commercial feed [250]. Studies have demonstrated that environments with sustained excretion of resistant determinants substantially alter the soil microbiome [10,251], furthering succession of MGEs at relatively low fitness costs [62]. Similar to routine antibiotic use, manure waste disposal in the same locations can function as a directional selection pressure on soil microbiota [252]. In Ecuador, AMR bacterial profiles of household and surrounding soil environments demonstrated strong associations suggesting shared selection pressures [119]. Antimicrobials administered in poultry farming remain the leading driver of AMR environmental pollution in Egypt [253,254]. It is speculated that *mcr-1,* originally detected in poultry production in China, is now globally present among livestock and humans resistomes [255,256,257,258]. Despite Chinese regulations to ban colistin, *mcr-1* remains present in the environmental resistome [149,252,259,260]. Furthermore, whole-genome sequencing has detected carbapenem-resistant *E. coli* among dogs, humans, flies, commercial poultry operations, and farmers [228]. The environmental resistome enables the persistence of AMR determinants across diverse hosts and demonstrates the role of environmental reservoirs.

Many mechanisms can facilitate the colonization of AMR bacteria or the transmission of ARGs into human microbiota through environmental resistome. Monitoring these dynamics within a low-resource setting is understudied [247,261]. Limited hygienic practices in combination with crowding can promote a high risk that environmental AMR bacteria can colonize human microbiota through a multitude of pathways including wildlife vectors, fecal-oral route, foodborne, contaminated water, or uptake from plants [30,262,263]. For example, there is evidence of genetic exchange among phylogenetically diverse organisms such as *Clostridium perfringens*, *Streptococcus pneumoniae, Enterococcus faecalis*, and strains of *Bacteroides* [264]. Studies have also recognized the potential for soil bacteria (e.g., *Burkholderia cepacia*, *Ochrobactrum intermedium*, and *Stenotrophomonas maltophilia*) to function as reservoirs for AMR [265]. 

Evidently, environment functions as a reservoir for active antibiotics, metabolites, and genetic material in the form of ARGs and MGSs [266,267,268]. Farm solid and wastewater can contaminate runoff that seeps into critical reservoirs of resistance including ground water, surface water, soil, and fertilizer [77,88,226,269]. Environmental contamination of antimicrobial residue might further lead to adverse human health effects, such as allergic hypersensitivity reactions, toxicity, nephropathy, mutagenicity, carcinogenicity, and AMR [270]. Furthermore, studies have demonstrated that ARGs can persist in soils for up to several years after chicken waste is removed from farm environments [19,251]. These findings suggest that reduction in AMU alone cannot effectively eliminate AMR bacteria from the environment. Comprehensive evaluation of environmental reservoirs in parallel with poultry waste removal is necessary for mitigating AMR emergence.

## 8. Barriers to Antimicrobial Stewardship Programs 

AMR, facilitated by antibiotic consumption, remains a dire global public health issue. Antimicrobial stewardship programs (ASPs) are curtailed by a variety interactive factors [140,271,272]. ASPs resemble a diverse makeup of system- and organizational-based interventions to address global AMR [147,273]. However, research and surveillance of AMR in low-resource settings is severely understudied [49]. Aquatic and terrestrial food animal production have intensified in the last decades to meet rapidly growing demands for quality sources of protein coupled with an expanding middle class and urbanizing population [129]. In these settings, vulnerable populations can be faced with multiple other drivers of morbidity and mortality [274]. Challenges to improved poultry antimicrobial stewardship programs (ASPs) extend into complex social and economic systems [190,275]. Evaluation of the ASP-inhibiting factors can inform decision making towards mitigating the impacts of AMR. Many LMICs have national plans to control AMR under a ‘One Health’ approach as encouraged by WHO, FAO, OIE, and regional institutions [15,276,277]. However, in-paper laws to regulate drug use in human and livestock are poorly enforced and surveilled, and particularly, interventions prescribing in animal health are scarce [278].

### 8.1. Limited Research and Surveillance

In resource-limited settings, disease surveillance typically captures a marginal understanding of the system. Surveillance is heavily hindered by the lack of resources and political commitment in supporting AMS agendas [279,280,281]. Many middle-income countries, especially within South America, fall short of delivering effective AMR surveillance due to inadequate political support [7]. Quantitative metrics of AMU within LMICs are not available [28]. On the other hand, international reports estimating presence of AMU exhibit high levels of variability due to the lack of standardization [28]. Effective interventions are dependent upon a more comprehensive understanding of AMU from established baselines [85]. Many LMICs lack trained personnel and resources to effectively monitor antimicrobial administration [116]. In many LIMCs, bacterial culture independent methodologies (e.g., partial or full genome sequencing) that allow screening drug resistance are not readily available due to high costs [140]. International standards for AMR surveillance are essential for monitoring antimicrobial use in poultry farming. Important projects carried out in LMICs depended on international collaborations with researchers coming from HICs. Moreover, global capacity is required to prevent the fast spread of AMR considering that international borders are crossed by over one billion people each year. AMR is a problem of a pandemic scale that should be better understood by veterinarians, farmers, policy makers, and the general public.

### 8.2. Misperceptions about Antimicrobial Resistance 

In LMICS, misperceptions of AMU and AMR emergence is common among small-scale poultry farmers [118,271]. This knowledge gap is further exacerbated by the fact that antimicrobials are typically purchased over the counter in animal agriculture stores [140,271,282]. Mekong Delta of Vietnam, 84% of poultry farms surveyed reported prophylactic rather than therapeutic AMU, and over 30% of antimicrobial classes administered were categorized of critical importance to human medicine according to the WHO’s priority list [48]. In Khartoum state of Sudan, approximately 50% of small-scale farmers lacked knowledge of common zoonotic diseases, and 30% were able to define AMR [283]. Similarly, a focus group of Peruvian veterinarians reported that inappropriate AMU is widespread and largely driven by many barriers including availability of antibiotics, competition with other veterinarians, economic constraints of farmers, and limited knowledge of animal diseases among farmers [284]. In the same sense, studies have recognized farmers as ill-informed on the functionality of antibiotics specific to bacterial infections and application of different antibiotic classes [285,286]. Improved access to quality veterinary services is necessary to alleviate misconceptions surrounding antibiotics in animal husbandry. 

### 8.3. Lessons Learned in Access to Veterinary Services

Quality veterinary services are fundamental to mitigating global emergence of bacterial resistance. Recent analysis conducted by the World Organization for Animal Health (OIE) reported that the majority of national veterinary services are suboptimal [116]. This finding presents an important biosecurity risk, not only to food production operations within many LMICs but also to the trading partners of these countries [190]. International aid support to LMIC veterinary services is very limited [116]. In 2006, OIE reported that 25% of African 50% of Middle Eastern countries lack national programs for mitigating animal disease outbreaks [287]. Maximizing profits can also negatively motivate veterinarians to excessively promote the use of antibiotics [116]. Furthermore, the majority of countries with animal health agendas were selective at addressing one or more specific pathogens [287]. One major gap is that many of these countries lack national educational curriculums [116]. Another obstacle to effective governance of veterinary services is political commitment. Often veterinary service policy related to antimicrobial use is outdated or nonexistent further limiting the efficacy of antimicrobial stewardship [190,206,288,289]. Post-market review of antimicrobial agents is almost nonexistent as fraudulent veterinary antimicrobial products regularly enter markets, leading to serious impacts of therapeutic efficacy [288]. Properly managed, transparent, and credible veterinary services are imperative for mitigating AMR spread.

## 9. Conclusions

The majority of antimicrobial use (AMU) is for food animal production [7]. Poultry encompasses the most abundant and fastest growing per capita livestock and one of the most common sources of multi-resistant (MDR) bacteria [35,36]. As countries continue to transition from low- to middle income countries (LMICs), a demand for quality sources of animal products will follow. Further promotion of intensive poultry farming could also address issues related to food security. In particular, special attention is needed among within the context of Brazil, Russia, India, China, and South Africa (BRICS); these nations encapsulate the majority of global livestock production and AMU [28,290,291]. Agricultural intensification is also a major driver for the emergence of antimicrobial resistance (AMR) and increasing the overall resistome. A systems framework is needed in order to reduce the burden of bacterial resistance within humans, animals, and the environment. 

Throughout the world, national veterinary service standards fall short of meeting international standards [116]. Access to trained veterinarian services can substantially improve diagnostic capability, treatment, and prescribed poultry antibiotic use. Investment in quality veterinary services is essential in two-part, to: (i) provide early detection and diagnostics of AMR and (ii) establish effective biosecurity and biocontamination measures [116]. As outlined by the World Organization for Animal Health (OIE), effective veterinary systems are critical for stabilizing economies, improving food security and food safety, and reducing exposure of AMR and pathogenic microorganisms [276]. Effective veterinary governance would not only reduce burdens of AMR but also simultaneously improve other infectious disease burdens [1,26,88,89]. Numerous countries have already systematically required veterinary services to oversee animal production, slaughter, food processing, product distribution, retail store inspection, and foodborne and occupational disease exposure surveillance programs [28,133,292]. Investment through capital and training could strengthen the capacity of veterinary services within LMIC food animal systems. Global food safety is not only an inherit concern of animal and public health, but also that of market viability for international trade partners. 

Effective veterinary services need to work in partnership with human medical services within a broader public health; many scientists have recognized this effort as a ‘One Health’ framework [9,139,293,294]. This approach instills a comprehensive approach for bolstering surveillance efforts across humans, animals, and the environment [295]. Various studies have reported that availability of veterinary services has strong potential for improving human and animal health as well as household income [296]. Within the last few decades, pandemics originated from animal reservoirs such as COVID-19 (SARS-CoV-2), Influenza A (H1N1), and West Nile Virus (WNV) have highlighted the necessity for public health interventions at the human-animal intersection in an effort to prevent zoonotic spillover events into human populations [297,298]. OIE conducted a review highlighting the cost effectiveness of preventive investments significantly surpass intervention costs [299]. For instance, restrictions on AMU across 17 nations could suggest reduction in antibiotics can be achieved without substantial impacts on productivity [300]. Moreover, a better understanding of the evolution of antibiotic resistance is needed to guide cutting-edge interventions. The implementation of research infrastructures and tracking systems (i.e. laboratory networks) is critical to collect data for decision-making and sharing data on AMR at a global level. Likewise, advanced molecular tools to identify ARGs, MGEs, and bacterial hosts are necessary to better understand transmission dynamics and evolution of AMR at human-livestock-environment interface. Even though the use of antibiotics in livestock is decreasing and “antibiotic-free” farms are becoming popular, the persistence of MDR bacteria in those animals constitutes a global concern. The efficacy of AMU reduction to control AMR was proposed due to studies showing that AMR implies a fitness cost, reducing bacterial growth rate and virulence. However, bacteria are evolving compensatory adaptations that reduce the cost of AMR. Therefore, reducing antibiotic use could have minimal effects in the short term on the poultry farms previously exposed to antibiotics. However, the AMU bans in HICs showed that the levels of resistance decreased in the long term [10].

The Global Action Plan on Antimicrobial Resistance endorsed by the member states of the WHO and affirmed at the high-level meeting on antimicrobial resistance during the 71st General Assembly of the UN [276], recommends that all countries collect and report antibiotic consumption data [23,28,160]. Within this doctrine, the WHO has established critically important antimicrobials for human medicine (WHO CIA list) [301]. Furthermore, the WHO CIA list consists of quinolones, cephalosporins (third and higher generations), macrolides and ketolides, glycopeptides, and polymyxins [301]. Administration of critically important antimicrobials to public health remains largely unregulated within low-resource regions [300]. It is critical that metrics of accurate antimicrobial consumption, both therapeutic and nontherapeutic use, are made available to monitor AMU within LMICs. In 2017, WHO requested affiliated nations to reduce veterinary AMU [85]. Researchers have already called to develop a standardized, internationally endorsed monitoring system for accurately collecting AMU data from food-production facilities [28]. Quantification of AMU in animal and human health is a primary goal of the Global Action Plan on Antimicrobial Resistance and related international plans and strategies designed by FAO, WHO, and OIE [276].

It has been widely accepted that LMICs require unique interventions compared to HICs due to their unique structural, cultural, and socioeconomic factors affecting AMR emergence [302]. AMR emergence [245,291,303,304]. Improved AMR surveillance by developing a standardized framework could lead to: (1) monitor consumption trends and establish goals for antimicrobial consumption, (2) provide a baseline of AMU consumption rates for comparison between countries at the scales of bacterial species, food animals, and human populations, (3) develop longitudinal studies determining the associations between antimicrobial consumption and AMR emergence [28]. In the case of China, a nationally instituted “ecological rationality” in regard to pig and poultry medium-scale operations improved biosecurity through more sustainable waste management [305].

The arsenal of antimicrobials administered in raising food animals is rapidly declining, while remaining essential for animal health, agrarian livelihoods, and public health. Careful evaluation of antibiotic use surrounding intensive poultry development could prevent further dissemination of drug resistance. Veterinary medicine implementations should target existing regions where resistance is emerging. Adopting sustainable poultry husbandry practices could lessen the rise or resistance. There is an obligation of all countries to improve stewardship of antimicrobials as an effort to improve biosafety and biosecurity. 

## Figures and Tables

**Table 1:** Summary of poultry production systems [93].

System	Housing	Characteristics
Broilers	Assumed to be primarily loosely housed on litter, with automatic feed and water provision	Fully market-oriented; high capital input requirements (including infrastructure, buildings, equipment); high level of overall flock productivity; purchased non-local feed or on farm intensively produced feed
Layers	Housed in a variety of cage, barn, and free-range systems, with automatic feed and water provision	Fully market-oriented; high capital input requirements (including infrastructure, buildings, equipment); high level of overall flock productivity; purchased non-local feed or on farm intensively produced feed
Backyard	Simple housing using local wood, bamboo, clay, leaf material and handmade construction resources for supports (columns, rafters, roof frame) plus scrap wire netting walls and scrap iron for roof. When cages are used, these are made of local material or scrap wire	Animals producing meat and eggs for the owner and local market, living freely. Diet consists of swill and scavenging (20–40%) and locally produced feeds (60–80%)

**Table 2:** Characteristics of family poultry production systems [15,103].

Criteria	Small-Extensive Scavenging	Extensive Scavenging	Semi-Intensive	Small-Scale Intensive
Production Operation	Mixed, poultry and crops, often landless	Mixed, livestock and crops	Usually poultry only	Poultry only
Other livestock raised	Rarely	Usually	Sometimes	No
Flock size	1–5 adult birds	5–50 adult birds	50–200 adult birds	>200 broilers >100 layers
Poultry breeds	Local	Local or cross-bred	Commercial, cross-bred or local	Commercial
Source of new chicks	Natural incubation	Natural incubation	Commercial day-old chicks or natural incubation	Commercial day-old chicks or pullets
Feed source	Scavenging; almost no supplementation	Scavenging; occasional supplementation	Scavenging; regular supplementation	Commercial balanced ration
Poultry housing	Seldom; usually made from local materials or kept in the house	Sometimes; usually made from local materials	Yes; conventional materials; houses of variable quality	Yes; conventional materials; good-quality houses
Access to veterinary services and veterinary pharmaceuticals	Rarely	Sometimes	Yes	Yes
Mortality	Very High; >70%	Very High >70%	Medium to High 20% to >50%	Low to Medium <20%
Access to reliable electricity supply	No	No	Yes	Yes
Existence of conventional cold chain	No	Rarely	Yes	Yes
Access to urban markets	Rarely	No, or indirect	Yes	Yes
Products	Live birds, meat	Live birds, meat, eggs	Live birds, meat, eggs	Live birds, meat, eggs
Time devoted each day to poultry management	<30 min	<1 hr	>1 hr	>1 hr

**Table 3:** Contributions of small-scale poultry to the UN Sustainable Development Goals [39,55].

Contribution Pathway of Small-Scale Poultry	Sustainable Development Goal
Increasing the availability, accessibility, utilization and stability of supply of food and nutrients.	2: Zero hunger 3: Good health and well-being
Small-scale poultry are able to be kept by vulnerable groups, giving them access to a source of income. Community-supported models for Newcastle disease prevention can provide employment, including for women, and increased production can promote rural economic growth.	1: No poverty 8: Decent work and economic growth
By targeting a livestock species and production system that is largely under the control of women, improvements to the SSP production systems can preferentially benefit women, promoting their empowerment. Income under the control of women is also more likely to be used to support the education of their children.	5: Gender equality 4: Quality education
Efficient and sustainable use of natural resources while achieving adequate nutrition globally requires high-income countries to decrease food wastage and consumption of calorie-dense, nutrient-poor foods, while low-and-middle-income countries need to increase their consumption of nutrient-rich foods. Small-scale poultry are nutritious and locally available, typically with a short supply chain, and measures to improve health and welfare will improve production efficiency and ensure sustainability.	12: Responsible consumption and production
Production of SSP does not require land clearing, contributes positively to ecosystem health, and can reduce loss of biodiversity by being a rich pool of genetic diversity and by being an alternate protein source to bushmeat.	15: Life on land

**Table 4:** Overview of antimicrobial resistance (AMR) transmission pathways originating from poultry production within resource-limited settings.

Country	Setting	AMR Transmission Pathway(s)	Operation Scale	Findings	Ref.
India	Urban	Intensive chicken farming	Large	High prevalence of multidrug resistance (94%) and ESBL-producing *E. coli* (87%).	[148]
Zimbabwe	Rural Urban Peri-urban	Intensive chicken farming	Small Large	Higher *Salmonella* spp. AMR levels with farming intensity. 12.1% MDR *S. enteritidis* isolates, presents public health risk of salmonellosis.	[102]
Kenya	Rural	Intensive chicken farming	Small Large	Documented drug-resistant thermophilic *Campylobacter* spp. originating in small-scale family operated poultry systems.	[149]
Nigeria	Urban	Cross-species AMR transmission	Large	High abundance of AMR and virulent *Enterococcus* spp. sampled from poultry and cattle manure suggesting spread between livestock species.	[150]
Ecuador	Rural	Cross-breed AMR transmission Zoonotic AMR transmission	Small	High increase (66.1%) in beta-lactamase CTX-M-producing *E. coli* of backyard chickens not fed antibiotics after the village-scale introduction of broiler chickens. Sequenced blaCTX-M demonstrated close relatedness of backyard chicken, broiler chicken, and human samples from the villages which could suggest AMR zoonotic transmission.	[139]
India	Rural	Indirect transmission to backyard poultry	Small	Detected high prevalence of MDR and avian pathogenic *E. coli* associated virulence genes 75.5% (n = 272) from backyard layer chickens and their environment. Potential AMR contamination from human defecation in nearby ponds and/or commercial broiler chicken flocks.	[151]
Ecuador	Rural	Indirect transmission to backyard poultry	Small	Reported thermophilic resistant *Campylobacter* spp. present in free-ranging backyard chickens that were not fed antibiotics.	[152]
Bangladesh	Urban	Intensive chicken farming Zoonotic	Large Medium	MDR presence in all *E. coli* isolated from intensive poultry, poultry husbandry environments, and hands of poultry workers.	[153]
Costa Rica	Rural	Transmission to wild birds	Small	Free-ranging poultry present a risk for transmitting resistant *E. coli* to neotropical avifauna.	[154]
Kenya	Rural	Indirect transmission to backyard poultry	Small	*E. coli* and *Salmonella* spp. were isolated and detected presence of class 1 integrons beta-lactamase genes from backyard chicken feces.	[155]
Vietnam	Rural	Intensive chicken farming Occupational exposure	Small Medium	Demonstrated an association with AMR *Salmonella* spp. in farmers and intensively farmed poultry.	[156]
E.U.		Zoonotic	N.S.	Human and food-production animals had moderate to high prevalence of *E. coli* and *Salmonella* resistant to ampicillin, tetracyclines and sulfonamides. High to extremely high resistance to fluoroquinolones in *Salmonella* spp., *E. coli* and *Campylobacter* recovered from humans, broilers, fattening turkeys and poultry carcasses/meet. Low levels of bacteria resistant to colistin in food-producing animals. MDR *Salmonella enterica* serotype Infantis recovered from broilers.	[157]
U.S.A.		Zoonotic	N.S.	High levels of *Campylobacter* resistant to ciprofloxacin in humans was associated to consume of raw or undercooked chicken, unpasteurized milk, contaminated food and water, and direct contact with animals. Moderate levels of *Salmonella* resistant to ciprofloxacin associated to direct and indirect contact with animal feces. MDR *Salmonella enterica* serotype Infantis recovered from broiler’s meet. Whole-genome sequencing revealed that this strain was identified from sick people returning from South America, and it is rapidly spreading among people and animal populations.	[158]
